# Detection of siRNA induced mRNA silencing by RT-qPCR: considerations for experimental design

**DOI:** 10.1186/1756-0500-3-53

**Published:** 2010-03-03

**Authors:** Katherine Holmes, Catrin M Williams, Elinor A Chapman, Michael J Cross

**Affiliations:** 1 Department of Pharmacology and Therapeutics, School of Biomedical Sciences, University of Liverpool, Liverpool, L69 3GE, UK; 2 NWCRF Institute, School of Biological Sciences, Bangor University, Bangor, LL57 2UW, UK

## Abstract

**Background:**

RNA interference (RNAi) has been one of the most rapidly expanding areas of biological research in the past decade, revolutionizing the ability to analyze gene function. Thorough validation of siRNA duplexes is required prior to use in experimental systems, ideally by western blotting to show a reduction in protein levels. However, in many cases good antibodies are not available, and researchers must rely on RT-qPCR to detect knockdown of the mRNA species.

**Findings:**

We have observed a phenomenon that gives a disparity between analyzing small interfering RNA (siRNA) efficacy by western blotting of the protein levels and real-time quantitative PCR (RT-qPCR) measurement of mRNA levels. Detection of this phenomenon was dependent upon the location of the target amplicon for PCR primers within the mRNA.

**Conclusions:**

Our data suggests that for certain mRNAs, degradation of the 3' mRNA fragment resulting from siRNA mediated cleavage is blocked, leaving an mRNA fragment that can act as a template for cDNA synthesis, giving rise to false negative results and the rejection of a valid siRNA duplex. We show that this phenomenon may be avoided by the careful design of RT-qPCR primers for each individual siRNA experiment.

## Background

RNA interference (RNAi) was first observed in *Caenorhabditis elegans *by Fire, Mello et al. [1], who found that introduction of double stranded RNA resulted in the silencing of gene expression. In the past decade RNAi has been one of the most rapidly expanding areas of biological research, allowing the development of RNAi as a therapeutic approach in the treatment of several disorders, including cancer and autoimmune disorders [2]. The RNAi pathway has also been utilized *in vitro*, enabling the knockdown of genes, and revolutionizing the ability to analyze gene function. The initial stage of analysis of gene function is to fully characterize the extent of siRNA mediated gene knockdown, as in many cases the gene expression is not completely inhibited. Knockdown of the mRNA is easily quantified using real-time quantitative PCR (RT-qPCR), whilst knockdown of the protein is visualized with SDS-PAGE and western blotting. Here we report upon a phenomenon which affects RT-qPCR quantification of gene knockdown, and which could result in false negative results, and the rejection of valid siRNA duplexes.

RNAi is initiated by the presence of long dsRNA molecules in the cell. These are cleaved into small-interfering RNA (siRNA) duplexes, 21-26 nucleotides in length, by Dicer, a member of the RNase III family of enzymes [3]. In mammalian cells, 21 nucleotide siRNAs can be introduced directly into the cell by transfection, in order to achieve gene silencing [4]. The siRNAs are incorporated into the RNA-induced silencing complex (RISC), targeting the complex to complementary mRNA substrates for degradation [5,6]. Degradation of mRNA by the RNAi pathway is then initiated by cleavage of the mRNA within the region complementary to the siRNA [7]. The fate of mRNA fragments generated by siRNA directed cleavage is not fully understood, but it is thought that they enter normal cellular mRNA degradation pathways [8]. For example, in Drosophila, the 5' fragment is degraded by the exosome, comprising 3'-5' exonucleases and the Ski complex, while the 3' fragment is degraded by the 5'-3' exonuclease XRN1 [9].

We have observed a phenomenon which may give rise to false negative results when assessing siRNA induced gene knockdown by quantitative real time-PCR (RT-qPCR). Since western blotting, the preferred method of detecting knockdown, cannot always be performed due to the current lack of antibodies to some genes, we propose the use of a primer design strategy to minimize observation of false negative results when using RT-qPCR to detect knockdown.

## Materials and methods

### Cell Culture

Human dermal microvascular endothelial cells (HDMECs) were purchased from Promocell (Heidelberg, Germany) and were cultured in endothelial cell basal media (EBM) MV2 growth media (C-22221; Promocell, Heidelberg, Germany), supplemented with 5% (v/v) fetal calf serum (FCS) and EGF (5 ng/ml), VEGF (0.5 ng/ml), FGF-2 (10 ng/ml), long R_3 _insulin growth factor-1 (20 ng/ml), hydrocortisone (0.2 μg/ml) and ascorbic acid (1 μg/ml) (supplement pack C-39221; Promocell, Heidelberg, Germany).

### siRNA transfection

siRNA duplexes were obtained from Qiagen (Crawley, UK). HDMECs were transfected with siRNA duplexes at concentrations of 1-10 nM using 0.1% (v/v) Lipofectamine RNAiMAX (Invitrogen, Paisley, UK), according to the manufacturer's instructions. Transfection reactions were performed in serum-free OptiMEM (Invitrogen, Paisley, UK). Cell media was changed to serum containing media 4 hours after transfection.

### RT-qPCR

**Figure 1:**
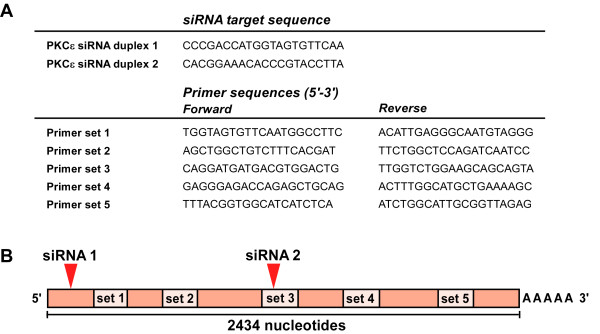
**Relative location of siRNA target sites and primer amplicons**. **(A) **Target mRNA sequence of each PKCε siRNA duplex, and sequences for each PKCε primer set. **(B) **Location of primer amplicons (set 1-5) in relation to the sites of siRNA directed cleavage (siRNA 1 and siRNA 2) of human *PKCε *mRNA.

Total RNA was extracted from HDMECs using the RNeasy mini kit (Qiagen, Crawley, UK), 24 and 72 hours post-transfection. DNase treatment was performed using the on-column DNase digestion (Qiagen, Crawley, UK). One μg total RNA was used for cDNA synthesis with M-MLV reverse transcriptase and either random hexamers or oligo(dT) (18T) primers. RT-qPCR was performed using Power SYBR Green Mastermix (Applied Biosystems, Warrington, UK). Primers were designed using the Invitrogen oligoperfect designer web tool, and were designed to give an amplicon of approximately 150 base pairs (figure 1A). Primer sequences were screened using a BLAST search to confirm specificity, and the PCR products run on an agarose gel to confirm that products of the expected size were detected. The efficiency of each primer set for RT-qPCR was determined to be between 95 and 100%. Alternatively pre-designed primers from Qiagen (Crawley, UK) were purchased. Reactions were analyzed upon an ABI 7000 real-time PCR machine using the following cycle conditions: 50°C for 10 minutes, 95°C for 10 minutes, followed by 40 cycles at 95°C for 15 seconds and 60°C for 1 minute. Results were normalized against *β-actin *expression.

### Immunoblotting

Protein lysates from HDMECs were prepared in LDS sample buffer containing 2.5% (v/v) β-mercaptoethanol. Proteins were resolved by SDS-PAGE on 4-12% NuPage Gels, and transferred onto nitrocellulose membranes (GE Healthcare, Bucks, UK). Membranes were blocked with 5% BSA. Antibodies to the C-terminus of PKCε and to actin were obtained from Millipore (Watford, UK) and Santa Cruz (Heidelberg, Germany) respectively. Membranes were washed 6 times with TBS-T, and incubated with peroxidase-conjugated secondary antibodies (GE Healthcare, Bucks, UK/Sigma, Dorset, UK). Blots were detected using an enhanced chemiluminescence (ECL) detection kit (GE Healthcare, Bucks, UK).

## Results and Discussion

**Figure 2:**
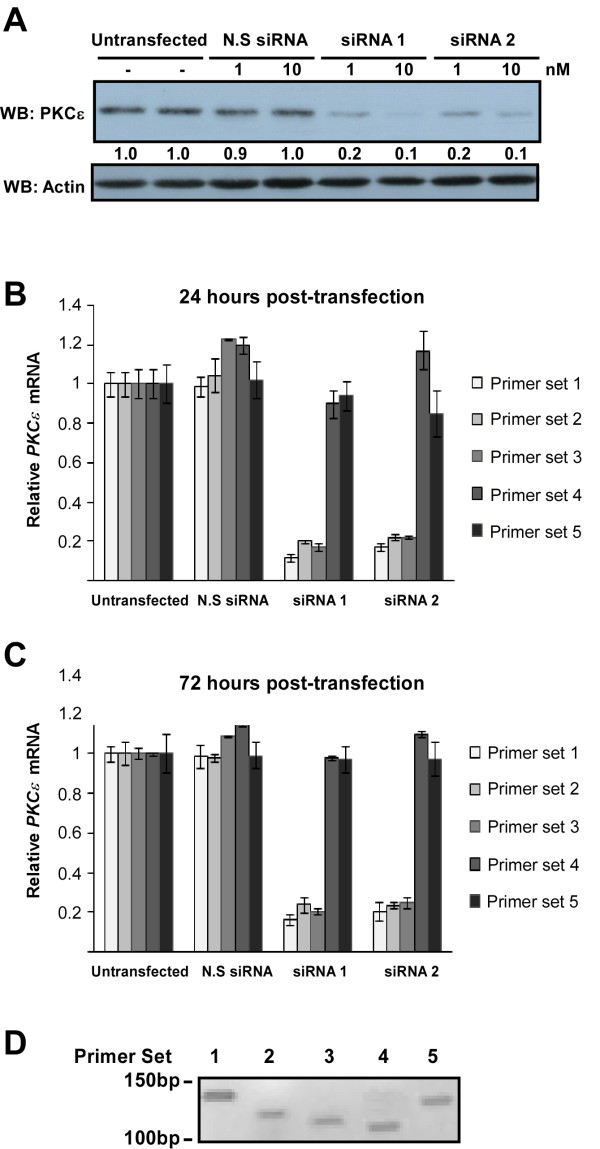
**Detection of PKCε knockdown by western blotting (WB) and RT-qPCR**. HDMECs were treated with two siRNA duplexes to PKCε, or with a non-silencing control siRNA (N.S siRNA) at either 1 or 10 nM and RNA or protein prepared at 24-72 hours post-transfection. **(A) **Protein lysates prepared 48 hours post-transfection were analyzed for PKCε expression by western blotting (WB). Numbers below the blot denote the relative intensity of each band. The detection of actin expression was performed to monitor protein loading. **(B) **The amount of *PKCε *mRNA transcript at 24 hours post-transfection was analyzed by RT-qPCR using 5 different primer sets (typical Ct values for each primer set in untransfected cells were as follows: set 1: 25.7, set 2: 26.04, set 3: 25.8, set 4: 25.43, set 5: 25.51). Relative mRNA expression was determined using *β-actin *control (n = 3, mean ± S.D.). **(C) **The amount of *PKCε *mRNA transcript at 72 hours post-transfection was also assessed by RT-qPCR (typical Ct values: set 1: 25.82, set 2: 26.01, set 3: 25.92, set 4: 25.51, set 5: 25.64). Relative mRNA expression was determined using *β*-actin control (n = 3, mean ± S.D.). **(D) **Each primer set was tested to confirm a single PCR amplicon, visualized by agarose gel electrophoresis.

Validated siRNA duplexes directed to protein kinase C-epsilon (PKCε) mRNA were used to knockdown PKCε expression in HDMEC. Two separate duplexes were used targeting separate regions of the target mRNA (figure 1A-B). A series of primers were designed to detect amplicons along the length of the PKCε mRNA transcript (figure 1B). Successful knockdown of PKCε was observed using either of the siRNA duplexes as seen by the reduced levels of protein (figure 2A). Data obtained from RT-qPCR analysis of mRNA levels following siRNA knockdown, 24 and 72 hours post-transfection, is summarized in figures 2B &2C. In the case of siRNA duplex 1, all 5 primer sets had been designed to detect amplicons 3' to the site of siRNA directed cleavage. However, only primer sets 1-3 were actually able to detect the knockdown. Primer sets 5' of the cleavage site for siRNA duplex 2 detected the knockdown (sets 1 and 2), as did flanking primers designed to amplify the region containing the siRNA target sequence (set 3). However, primers designed to amplify sequences 3' of the cleavage site did not detect the knockdown (sets 4 and 5). There was no difference observed between data obtained using either oligo(dT) primers or random hexamers for cDNA synthesis (data not shown), confirming that this effect was not due to the cDNA priming method; oligo(dT) primers bind to the polyA tail of mRNA, while random hexamers bind to multiple sites along the mRNA molecule. We also confirmed that all the primer sets produced only a single PCR amplicon of expected size by running the products on an agarose gel (figure 2D).

Our data indicates that the location of the PCR amplicon could have implications for the validation of siRNA knockdown. Primers that were used to detect amplicons close to the 3' end of the mRNA did not detect the knockdown, whilst primers used to detect regions closer to the 5' end of the mRNA successfully detected the knockdown. We propose that degradation of the 3' mRNA fragment resulting from siRNA mediated cleavage is blocked, leaving an mRNA fragment that can act as a template for cDNA synthesis. The cause of this disruption is unknown, but could be related to the presence of RNA binding proteins, or due to the secondary structure of the mRNA. It has previously been reported that siRNA treatment can result in the accumulation of 3' mRNA cleavage products within the cell [10]. The authors suggest that this is due to the saturation of mRNA degradation pathways due to the rapid rate of the initial cleavage step, performed by the RISC complex. However, one would expect to see the mRNA cleavage products degraded within 24-48 hours post-transfection [11], whereas we were still able to detect PCR amplicons with primer sets 4 and 5 at 72 hours post-transfection (figure 2C). In addition, screening of other classical (PKC-α) and novel (PKC-δ, PKC-η) members of the PKC family using validated siRNAs and by designing primers along the length of their mRNA, did not reveal the same phenomenon (data not shown). *PKC-α *mRNA was expressed at five-fold higher levels than *PKC-ε *mRNA, whereas *PKC-δ *and *PKC-η *mRNA were expressed at very similar levels to *PKC-ε *(data not shown). Taken together, this data suggests that the observed phenomenon is unlikely to be due to saturation of the mRNA degradation pathways due to *PKCε *mRNA being highly expressed.

The fact that this phenomenon was not observed in all of the genes tested suggests that it is not common to all siRNA cleaved mRNA. However, the inability of certain primers to measure siRNA mediated mRNA degradation by RT-qPCR has been observed for connective tissue growth factor (*CTGF*) gene expression in a transformed human trabecular cell line [12], suggesting that this phenomena could occur for a number of genes. Our data, using primary human cells, confirms that this phenomenon can occur in non-transformed cells and has the potential to occur during *in vivo *delivery of siRNA.

Our finding has implications for the design of RT-qPCR experiments where primers are designed to generate short amplicons of 100-200 bp, typified by SYBR green and TaqMan methodology. Based on our observations we would recommend that, where possible, primers should ideally be designed to flank the siRNA target sequence. This approach would be a reliable method of ensuring that the initial cleavage of the mRNA could be detected. However, due to the thermodynamic constraints placed on primer design for use in RT-qPCR experiments, it is not always possible to design primers that will flank such a specific region of the mRNA. In this case it would be advisable to design primers which would detect regions within the 5' cleavage fragment, and couple this with either oligo(dT), or anchored oligo(dT) cDNA priming. If the siRNA has successfully cleaved the mRNA within the target region, then even in the presence of stabilized mRNA fragments, oligo(dT) priming would only result in a truncated cDNA species due to the cessation of cDNA synthesis at the siRNA target site. Regions 5' to the cleavage site would therefore not be amplified in a PCR reaction.

## Conclusions

We report for the first time a clear disparity between analyzing siRNA efficacy by western blotting of the protein levels and RT-qPCR measurement of mRNA levels. Ultimately the best way to confirm successful knockdown of a target gene by siRNA is to perform a western blot. However, if this is not possible RT-qPCR can offer an alternative approach, as well as allowing the extent of the knockdown to be quantified. Here we show that the design of primers for RT-qPCR experiments is an important consideration, as using a primer set targeted to the wrong region may result in false negative results, leading to the rejection of a valid siRNA duplex.

## Abbreviations

cDNA: complementary DNA; M-MLV: Moloney murine leukemia virus; mRNA: messenger RNA; PKCε: protein kinase C-epsilon; RNAi: RNA interference; RT-qPCR: real-time quantitative polymerase chain reaction; siRNA: small interfering RNA.

## Competing interests

The authors declare that they have no competing interests.

## Authors' contributions

KH performed the majority of the lab work profiling siRNA knockdown of PKC-ε and western blotting and co-wrote the paper. CW and EAC designed and tested PCR primers and validated siRNA duplexes. MJC conceived of the study, participated in its design and coordination and helped to draft the manuscript. All authors read and approved the final manuscript.
